# Self assembly of model polymers into biological random networks

**DOI:** 10.1016/j.csbj.2021.02.001

**Published:** 2021-02-12

**Authors:** Matthew H.J. Bailey, Mark Wilson

**Affiliations:** Physical and Theoretical Chemistry Laboratory, South Parks Road, Oxford OX1 3QZ, United Kingdom

**Keywords:** Continuous random network, Self assembly, Polygon statistics, Polygon distributions, Collagen network

## Abstract

The properties of biological networks, such as those found in the ocular lens capsule, are difficult to study without simplified models. Model polymers are developed, inspired by “worm-like” curve models, that are shown to spontaneously self assemble to form networks similar to those observed experimentally in biological systems. These highly simplified coarse-grained models allow the self assembly process to be studied on near-realistic time-scales. Metrics are developed (using a polygon-based framework) which are useful for describing simulated networks and can also be applied to images of real networks. These metrics are used to show the range of control that the computational polymer model has over the networks, including the polygon structure and short range order. The structure of the simulated networks are compared to previous simulation work and microscope images of real networks. The network structure is shown to be a function of the interaction strengths, cooling rates and external pressure. In addition, “pre-tangled” network structures are introduced and shown to significantly influence the subsequent network structure. The network structures obtained fit into a region of the network landscape effectively inaccessible to random (entropically-driven) networks but which are occupied by experimentally-derived configurations.

## Highlights

• Biological networks are hard to study without simplified computational models.
• Model polymers are shown to assemble into networks like those observed in biology.
• Coarse-grained models allow simulation of a self assembly process over microseconds.
• Metrics are developed to describe simulated networks and images of real networks.
• Tangling in the model polymers is shown to influence the network structure.

## Graphical abstract

**Figure:**
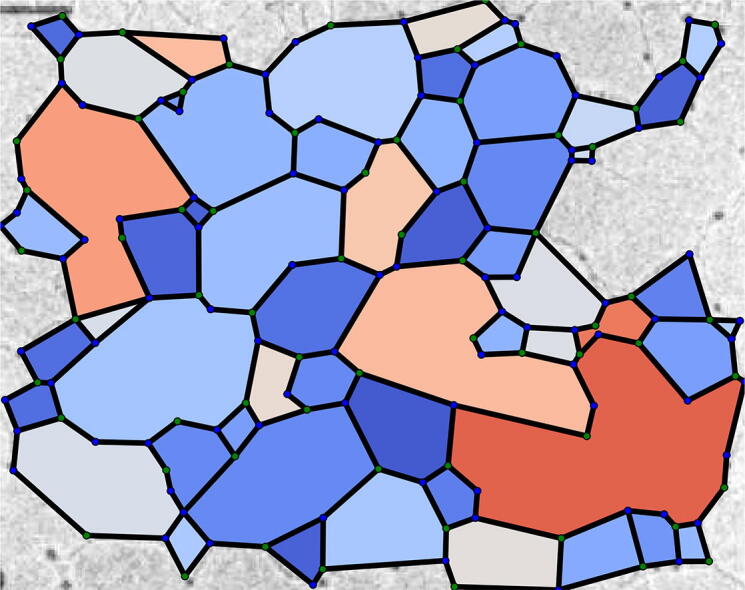

## 1 Introduction

Two-dimensional (2D) networks are critically important in biology — from basement membranes surrounding muscles, to the lens capsule of the eye. Furthermore, some three-dimensional (3D) networks are composed of stacked layers of 2D networks in which the intra-layer interactions are significantly larger than those of the inter-layer. The ubiquity of these systems makes understanding the formation and ageing of the 2D networks a key research interest, often with the aim that an understanding of the structure and properties of biological networks can lead to the development of synthetic materials that mimic natural biological networks. By understanding the millions of years of design experience nature has applied, stronger or stretchier materials can be developed allowing, for example, prosthetic replacements for parts of the body to be built [1].

One example of a 2D biological network is the collagen IV network in the ocular lens capsule. Artificial lens capsules can be implanted after cataract surgery, which can improve the ability to accommodate focal depths in elderly people [2]. The loss of focal accommodation in human senescence has been linked to collagen IV networks becoming less stretchy as they age [3]. For this reason, artificial intraocular lenses can often be superior to the removed biological lens [4]. The mechanism of this network ageing is a poorly-understood process, despite being a topic of scientific interest for 100 years [5].

There is a clear need for simplified computational models to study this network, as individual collagen molecules have backbones of over 1000 amino acids [6], and the ageing process occurs over a human lifetime [5]. These properties make it difficult to observe the relevant pathway, because it cannot be directly observed on laboratory timescales and the complexity of the individual molecules makes large-scale atomistic simulation computationally unfeasible. A further issue arises because the 2D networks are most interesting when they are part of living creatures. The removal of biological networks (and their preparation for study) can often disturb their delicate structure, with significant knock-on effects for their properties [7]. Previous work has used animal models, such as mice, cows, and monkeys, to understand the ageing of human lens capsules; or coarse-grained finite-element models to treat the whole lens as an engineering problem [8], [9], [1]. Previous computational models have been based on graph theory; these include Erdös-Rényi random graphs, Mikado networks and bond-switching of ordered graphs [10], [11], [12]. These graph approaches are parameterised and based on experimental data and small-scale simulations, but provide an incomplete link between detailed molecule simulations and the wider network behaviour.

Collagen IV molecules can spontaneously self-assemble into networks *in vitro*, which can occurs at more computationally accessible timescales than aging does [13], [14]. These self assembly studies have shown that the network structures formed are strongly dependent on the environmental conditions, for example on the concentration of dissolved salts [15], [16]. Such analysis provides some insight into which network arrangements are favourable, and how they self-assemble.

This work presents a highly simplified computational model for collagen-like molecules that self assemble into 2D network structures. The models are based on information obtained from experimental images of collagen IV networks, both self-assembled and in the ocular lens. The model is simple enough to be able to capture long timescales as well as to incorporate different interaction types.

First, a method of analysing 2D networks is established, including a way to unambiguously assign polygon structures. Next, biological random networks (both experimental images and simulated networks) are compared and contrasted to inorganic chemical networks, which have been studied using similar frameworks and which are generally more well-defined in terms of supporting a single atom local coordination number. The regularity of the polygons in the networks are a key point of comparison. Following that, the available range of control over the generated network structure is demonstrated by varying physically meaningful parameters, including pressure, cooling rates and the strength of attractions (a proxy for physical effects such as salt concentration). Finally, the generated networks are placed in the context of the range of experimental data available from microscope images of 2D collagen IV networks, and are shown to be more appropriate for reproducing these experimental networks when compared with previous continuous random (entropically-driven) networks.

## 2 Methods.

### 2.1 Network structures

Network theory has been long used to analyse biological structures ranging from hexagonal arrangements of cells to graph models of biopolymers [17], [10]. The existence of such a wide range of network models indicates that it is first helpful to establish a method to describe 2D networks rigorously, here using the gaps between molecules instead of the molecules themselves. Intuitively, a 2D network can be considered as being assembled from a collection of edge-sharing polygons — for example, a honeycomb is a network of hexagons, and chain-link fences may form a network of squares [18]. This concept can be extended to cover generic 2D networks across many length scales, from the atomic structure of glasses to the cantons of Switzerland [19], [20]. Viewing a 2D network as being constructed of polygons allows use of consistent metrics, applicable across many different types of network including those generated via a self assembly model, graph theory or experimental microscope images.

For example, the preference of polygons to be adjacent to similar or dissimilar polygons (described, for example, by the assortativity, and first discussed by [21] to describe social networks [21]) describes the short-range structure of a network and improves on the Aboav-Weaire parameter traditionally used to describe chemical 2D networks [22], [20]. Alternatively, the number of edges of a polygon (often referred to as their size) or their area is useful to quantify the voids in a network. In a biological context these voids could be affected by the presence of a scaffold, such as laminin [23], or by repulsive cations that the network forms around akin to a Voronoi partition [16]. A significant feature of biological networks (compared with inorganic glasses) is the ability to support a distribution of local coordination environments. The coordination number (called the *degree* in network theory) *k* of nodes in the network can be used to analyse the preference for different types of bonding between molecular head groups or lateral interactions.

There are numerous ways to describe the polygon structure of a network, and each has its own benefits and shortcomings. These include polygons chosen by shortest path criteria, primitive rings that cannot be decomposed into smaller rings, and a series of stronger criteria. Polygon (or “ring”) assignment criteria are discussed in great detail in Yuan and Cormack [24], and also in Le Roux and Jund [25]. The latter discusses their implementation in the R.I.N.G.S. code, a popular package for obtaining ring size distributions. For example some methods of assigning polygons to a network ensure each edge is belongs to one polygon, or the area is covered by a layer of polygons with no overlaps, or all shortest paths between nodes are included [24]. We have found these methods to be difficult to interpret, especially in the presence of nodes with k=2or in the case of small periodic systems.

**Fig. 1:**
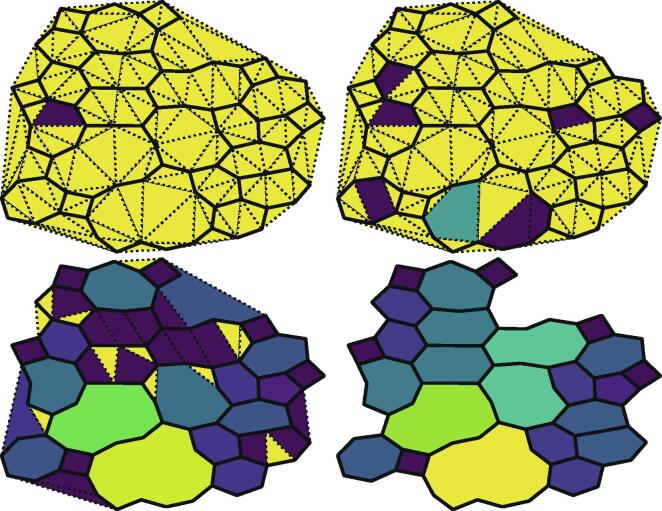
Snapshots from the process of constructing the polygon graph. Dashed edges are those that only exist in the Delaunay triangulation (top left), which are removed one-by-one (top right then bottom left). Removing a dashed edge merges two polygons together, and the final network structure (bottom right) is left after all dashed edges are removed. The polygons are coloured by their number of edges.

To this end, we have developed a method to describe the polygon structure utilising a Delaunay triangulation, a common technique to break large polygons into triangles often used in computer graphics applications. First, a network graph is created, in which small clusters of head groups are nodes, joined by edges representing molecules. An algorithmic description of this process can be found in the Supplementary Information Section I. The Delaunay triangulation of a set of nodes creates a set of triangles, whose edges connect neighbouring nodes and cover the entire area. We restricted our study to only networks where the Delaunay triangulation is a superset of the edges in the network; this criterion was met for all imaged and simulated networks we encountered. It is computationally simple to identify a triangle as a simple polygon. From there polygons with more edges can be built up by connecting smaller polygons, as shown in Fig. 1. If edges that are not in the original graph are systematically removed, updating the polygons at each stage, it is possible construct a set of unique polygons for the network that covers the entire area and maps neatly to an intuitive definition of polygons.

### 2.2 The simplified model

**Fig. 2:**
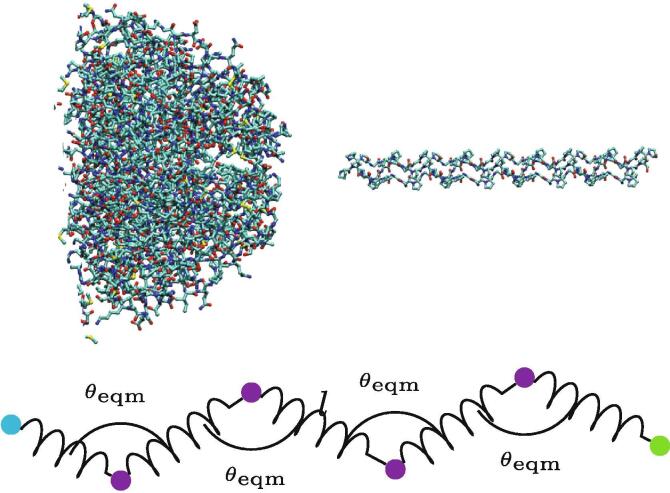
Two schematic parts of a collagen IV molecule, representing the simplification in the coarse graining process. The left-hand image represents a trimer in an NC1 domain, approximately 5000 atoms which are simplified into a single green head bead in the polymer representation [31]. The right hand image (shown on a different scale) shows one representative section of a triple helix joining the two ends of the collagen IV molecule, which is represented by two purple body beads and a joining spring [32]. The blue head bead at the other end of the polymer represents a 7S domain, which is not pictured here.

The assembly of collagen-like molecules was simulated using a model inspired by a combination of “worm-like” curve polymers and patchy particles. Patchy particles are colloids with attractive regions on their surface which have been shown experimentally and computationally to self-assemble [26], [27], and mimic the crystallisation properties of proteins [28]. The simplified collagen like molecules are represented by a series of beads joined by springs. Each pair of beads is joined by a stiff harmonic potential, and each adjacent trio of beads has their angle constrained by an angular potential as shown schematically in Fig. 2. This is a coarse-graining of a worm-like curve model, which have been used successfully in modelling the stiffness of biopolymers [29], [30]. The energy terms are controlled by the force constants, $k_{l}$ and $K_{\theta }$, respectively. The beads may have two (or more) types, here body (B) beads and head (H) beads. To mimic the effective excluded volume of a polymer biomolecule, the body beads repel each other according to a cut-off Lennard-Jones potential described by an energy scale ${∊}_{\mathrm{BB}}$ and a range $\sigma_{\mathrm{BB}}$

(1) $$U_{\mathrm{BB}}(r)=\left\{\begin{array}{cc} 4{∊}_{\mathrm{BB}}\left[{\left(\frac{\sigma_{\mathrm{BB}}}{r}\right)}^{12}-{\left(\frac{\sigma_{\mathrm{BB}}}{r}\right)}^{6}\right] & \;r<\sigma \\ 0 & \;r⩾\sigma \text{.} \end{array}\right)$$

Two head groups interact with one another, according to a Lennard-Jones potential with an energy scale ${∊}_{\mathrm{HH}}$ and a range $\sigma_{\mathrm{HH}}$ (representing an effective size of the bead),

(2) $$U_{\mathrm{HH}}(r)=4{∊}_{\mathrm{HH}}\left[{\left(\frac{\sigma_{\mathrm{HH}}}{r}\right)}^{12}-{\left(\frac{\sigma_{\mathrm{HH}}}{r}\right)}^{6}\right]\text{.}$$

$\sigma_{\mathrm{BB}}$and $\sigma_{\mathrm{HH}}$ are chosen to prevent molecules from interpenetrating and to favour k=3 coordination. Body-head interactions are set to zero throughout ($U_{\mathrm{BH}}=0$). There is only one type of head group interaction in this model, but real collagen IV molecules have been observed to have two different head groups: 7S and NC1. The 7S and NC1 domains interact only with other domains of the same type, and favour different coordination numbers, *k*. However, initial simulations with two types of head groups lead to networks with only even sized polygons forming; this is similar to the geometrical frustration observed in ferromagnetic systems. By changing the attractive part and range of the Lennard-Jones potentials, the screening effect of salts in the self assembly solvent can effectively be mimicked without explicitly taking them into account (which greatly reduces computational expense).

The solvent was taken into account implicitly using a Langevin thermostat, which sets the force $\overset{\to }{F}$ on the molecules to

(3) $$\overset{\to }{F}={\underset{︸}{{\overset{\to }{F}}_{\mathrm{Bond}}+{\overset{\to }{F}}_{\mathrm{Angle}}+{\overset{\to }{F}}_{\mathrm{LJ}}}}_{\mathrm{ConservativeForces}}-{\underset{︸}{\frac{m\overset{\to }{v}}{\gamma }}}_{\mathrm{Drag}}+{\underset{︸}{\xi \sqrt{\frac{k_{B}\mathrm{Tm}}{\gamma dt}}}}_{\mathrm{Brownian}}$$

This thermostat has the traditional conservative forces including bond forces ${\overset{\to }{F}}_{\mathrm{Bond}}$, intra-molecular angle forces ${\overset{\to }{F}}_{\mathrm{Angle}}$ and head group forces ${\overset{\to }{F}}_{\mathrm{LJ}}$, but adds a drag term featuring the mass *m*and velocity $\overset{\to }{v}$ of each bead, divided by a damping factor γ representing the viscosity of the solvent. Finally, the thermostat features a Brownian dynamics term with dt the timestep, *T*the desired temperature, and ξ a random number recalculated every step. In Section 3.4.2, the thermostat desired temperature *T*is changed over a period of time denoted $t_{\mathrm{cool}}$.

### 2.3 Simulation protocol

This work used LAMMPS to perform Molecular Dynamics simulations due to the ease of use and ready support for 2D systems [33]. Molecular Dynamics methods are well suited for studying self assembly methods, as they repeatedly numerically solve Newton’s equations of motions and thus naturally capture dynamic behaviour of a complex system. Unless otherwise mentioned, each set of parameters was sampled in ten different configurations of 20×20 molecules in a periodic simulation cell, initially placed on a square grid. The grid arrangement was equilibrated by simulation at T=30K for t=40μs, with an initial t=4μs limiting the maximum motion per timestep to prevent the simulation exploding. The drive to form a network was so strong that an initial network was commonly formed during equilibration. Next, the simulation cell size was relaxed using an *NpT* ensemble (*i.e.* constant number of particles, pressure, and temperature with the temperature controlled by a Nosé-Hoover thermostat instead of the Langevin thermostat) at T=30K with p=20Pa for a further t=40μs. This pressure was chosen based on parameter scans (discussed more in Section 3.4.3) as it best reproduced the biological networks of interest. The use of pressure is a shorthand, as the corresponding physical property is more accurately stress. However, LAMMPS treats 2D systems as being in a 3D cell with the *z*component of position and velocity set to 0. Following equilibration of the pressure, the simulation cell size was fixed and the simulation returned to a *NVT* (*i.e.* constant number of particles, volume and temperature) ensemble. The proto-network was heated to 300K by adjusting *T*in Eq. (3) over 100 μs, which melted it. This is a relatively low melting temperature for computational efficiency, and all energy values in the simulation could be arbitrarily rescaled if necessary. After melting was complete (as evidenced through equilibration of the structural metrics, *i.e.* k=1 for all molecules), the collagen polymer liquid was cooled over a final 100 μs to 30 K. The total time from T = 300 K to 30 K was recorded as the value $t_{\mathrm{cool}}$. Snapshots were extracted and analysed at the end of each simulation.

In general, only one parameter was varied across each set of simulations, with the remaining parameters fixed at the default values that most reliably produced networks (chosen after an initial scan of the effective parameter space). These default parameters were bond and angle energy scales as $k_{l}=1.657\times 10^{-4}\;{\mathrm{Nm}}^{-1},K_{\theta }=200\times 10^{-21}\;J$, a cooling time of $t_{\mathrm{cool}}=100\;\mu s$, Lennard-Jones head-head and body-body energy scales were ${∊}_{\mathrm{HH}}=4.142\times 10^{-21}\;J,{∊}_{\mathrm{BB}}=16.142\times 10^{-21}\;J$. The length scales in the simulation were such that the equilibrium polymer length was $l_{\mathrm{eqm}}=300\;\mathrm{nm}$, and the Lennard-Jones length scales were $\sigma_{\mathrm{HH}}=50\;\mathrm{nm}$ and $\sigma_{\mathrm{BB}}=137.5\;\mathrm{nm}$. The polymer length is similar to the observed length of collagen IV molecules, which has been reported to be in the range 300nm to 400nm [34], [35]. The body bead interactions are considerably larger than the actual width of a collagen IV triple helix, 1.5nm, as they also take into account the excluded volume effect of polymers where close coordination in entropically unfavourable. The Lennard-Jones length scales were chosen to favour k=3 coordination geometrically akin to the design of patchy particles. These parameters were chosen to promote the assembly of the coarse grained units into networks that best matched observed images, while avoiding “over-fitting” to the relatively sparse experimentally observed macroscopic properties. The parameters used in this work are generic by choice, and aim to reproduce as wide a variety of network types as possible, reflecting the variance in microscope images of biological networks — for example, images of collagen IV networks can vary dramatically depending on the environmental conditions or self assembly conditions [34], [36], [15], [14]. Future refinements of this model could more specifically reproduce properties of interest of a given biological network; for example, choosing $k_{l}$ to reproduce mechanical properties or to assign the stiffness of the angular potential based on experimental data as has been highlighted for chromosomes [37], [38].

## 3 Results and discussion

The polymer model described has been developed so as to be deliberately generic in order to capture as wide a range of biological network structure as possible. Current images of biological networks show an amazing diversity of form, structure and function. A number of previous approaches have been used to explore biological networks, including entropically driven random network methods and modelling random networks as being idealised structures. We compare the polymer network model to those different approaches before presenting the degree of control and improvements available in the proposed model.

### 3.1 Comparison to the ideal (maximum entropy) networks

A key feature of the polymer model is that it can take enthalpic effects into account naturally. The importance of enthalpy to biological networks can be demonstrated by comparing with polygon networks which are dominated purely by entropy, such as those studied using bond switching methods [12]. A simple, numerical model is the maximum entropy distribution. This maximises the entropy of polygon edge counts according to the following constraints [39], [20]:

(4a) $$\underset{n}{\sum }p_{n}=1$$

(4b) $$\underset{n}{\sum }{\mathrm{np}}_{n}=\langle n\rangle$$

(4c) $$\underset{n}{\sum }\frac{p_{n}}{n}=\mathrm{constant}$$

with $p_{n}$ the fraction of polygons with *n* edges. This system of equations can be solved numerically with Lagrange’s method of undetermined multipliers.

**Fig. 3:**
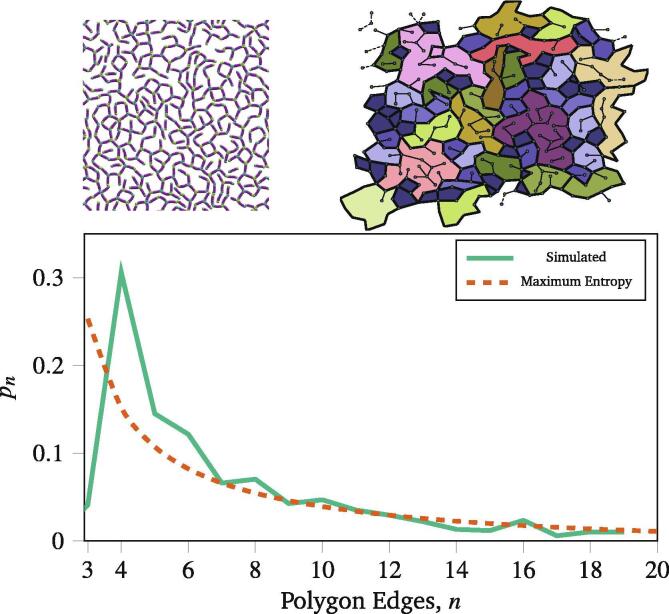
The process of applying network statistics to a network generated by the coarse graining procedure, here with the metric being polygon edge counts. First, a snapshot of a coarse grained simulation is taken (top left panel) and the polygons are identified (top right panel). Finally, the fraction of polygons with *n*edges (denoted $p_{n}$) is compared to the distribution predicted by maximum entropy (red dashed line).

The self-assembled networks show polygon edge count distributions which do not resemble the numerical maximum entropy model; this is visible in Fig. 3, which compares polygon edge counts from a polymer simulated and the polygon edge counts from maximum entropy model distribution. This was based on simulations described in Section 3.4.1 at $k_{l}=1.5\times 10^{-4}\;\mathrm{Nm}$. Similar differences are observed across all simulations, and the difference between a maximum entropy model and the distribution of edge counts from polymer simulations is sufficiently pronounced that the numerical fitting procedure often fails. For polygons with many edges, their population is accurately predicted by a maximum entropy approach because they are sufficiently flexible to minimise bond and angular strain. However, for polygons with few edges, such as triangles and squares, this angular strain is unavoidable. There are therefore very few triangles in the generated networks because of the strain involved in forming them, but an overpopulation of squares compared to the maximum entropy population.

### 3.2 Comparison to previous network studies

Prior to considering how different controllable variables may affect the network structure, a clear set of metrics is required. These metrics must capture the key differences that biopolymer networks exhibit compared with inorganic networks, describe the effects of enthalpy and entropy, and provide simple proxies for complex physical phenomena. These metrics build on those previously applied to characterise 2D networks [12].

#### 3.2.1 Polygon convexity

One interesting difference that shown by biopolymer networks, compared with inorganic networks is that the polygons are more likely to be concave or distorted. The strong angular potentials and fixed coordination numbers of atoms in inorganic glasses, such as silica, lead to a strong preference for convex polygons. This is not the case for polymer networks, which can have variable coordination numbers, more flexible angular potentials and even curved edges. To quantify this difference in shape regularity, we make use of a metric originally developed for computer graphics [40]. The shape regularity coefficient (*SRC*) of a shape S, is defined as

(5) $$\mathrm{SRC}(S)=\mathrm{SO}(S)V_{\mathrm{xy}}(S)\mathrm{CO}(S)$$

in which SO(S)is the solidity, defined as the ratio of the polygon area to the area of its convex hull; $V_{\mathrm{xy}}(S)$is the balanced repartition of the shape, defined as $\sqrt{\frac{\min(\sigma_{x},\sigma_{y})}{\max(\sigma_{x},\sigma_{y})}}$where $\sigma_{x(y)}$ is the standard deviation of the x(y) coordinate of the vertex positions; and finally CO(S)is the convexity, defined as the ratio of the convex hull perimeter to the polygon perimeter.

**Fig. 4:**
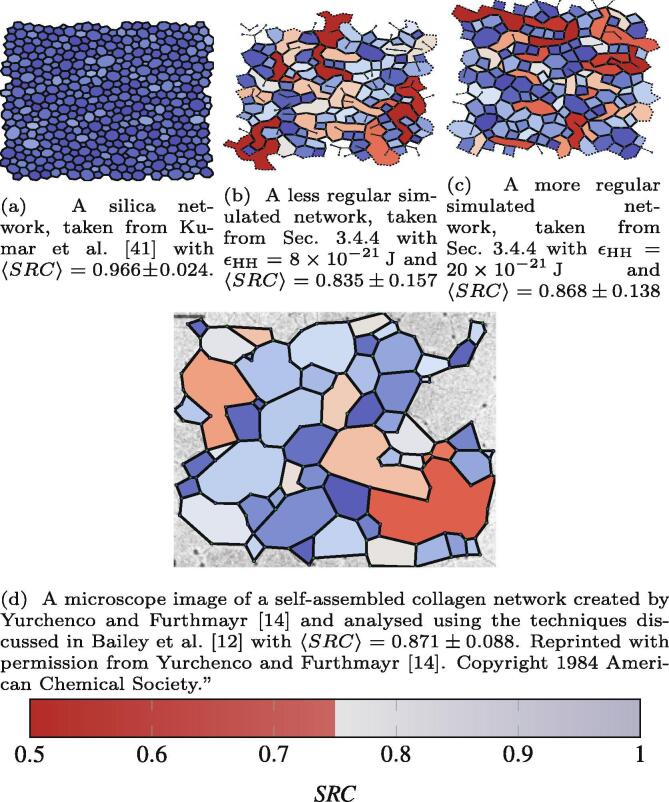
A comparison of an inorganic network Fig. 4a and two simulated biological networks [Fig. 4b and Fig. 4c]. The color ramp of red to blue represents the range of irregular (SRC=0.5) to regular (SRC=1) polygons.

Regular shapes, like a square, pentagon or circle are characterised by SRC=1. Less-regular shapes, such as ellipses or rectangles, have 0⩽SRC<1. Fig. 4 shows four example networks with varying degrees of regularity as defined by the *SRC*, with each polygon coloured by *SRC*. The silica network in Fig. 4a [41] shows highly regular polygons, corresponding to 〈SRC〉=0.966±0.024, the latter figure being the standard deviation. A more complete table of data can be seen in Table SI 1 of the Supplementary Information. The silica networks show 〈SRC〉≈0.96 with relatively narrow distributions (small standard deviations). By comparison biological networks show 〈SRC〉values which are significantly smaller. For example, networks generated in the present work show 〈SRC〉 in the range 0.747⩽〈SRC〉⩽0.928 (see Table SI 2). In a previous publication a number of experimental images were analysed [12]. The microscope images of collagen IV networks showed that the polygons formed were often irregular.

Table SI 1 lists the values of 〈SRC〉 obtained from 21 such images which show values in the range 0.69<〈SRC〉<0.91. As a visual example, Fig. 4d shows a section of a network from Yurchenco and Furthmayr [14] which corresponds to 〈SRC〉=0.871±0.088. The greater flexibility of the biological networks, compared to the silica, is highlighted by the broader range of *SRC* values observed in the former.

Overall, therefore, the flexibility of biopolymers leads to less regular polygons in a 2D network, and this matches observation from microscope images. Other factors that lead to lower *SRC*values are more flexible angular terms around nodes in the graph compared to inorganic networks, and the presence of k=2 sites which can lead to concave shapes. These concave shapes can be seen in Fig. 4b and Fig. 4d as the long shapes, coloured pale. In comparison, all polygons in Fig. 4a are convex and approximately isotropic. A full table of data with 〈SRC〉 values is available in the Supplementary Information as Table SI 2.

#### 3.2.2 Coordination number and internal energy

The energetics of the networks generated by the polymer self-assembly model, while interesting, are highly dependent on the potential energy of interaction between polymers. The short-range nature of the Lennard-Jones potential used in this work means that it is possible to use the node coordination numbers *k*as a proxy for the internal energy in the network. This removes the complicating effects of bond stretches and angular strain, and solely represents the energy gained in forming the network. This makes it possible to study the energetics of the network easily, using only the final polygon structure.

**Fig. 5:**
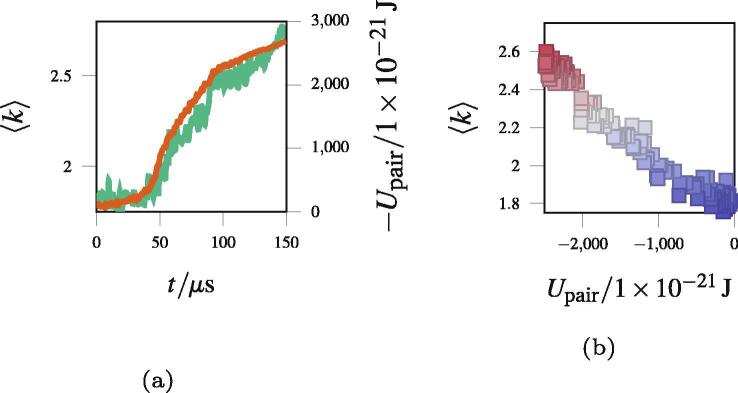
Two figures to highlight the strong correlation between the mean coordination number 〈k〉 and the pairwise head-group interaction energy $U_{\mathrm{pair}}$. Fig. 5a shows 〈k〉 as a function of time for a system undergoing network self assembly. Fig. 5b shows a scatter plot of 〈k〉 against $U_{\mathrm{pair}}$ to emphasize their correlation. Since 〈k〉 dominates the internal energy, we can use 〈k〉 as a simple proxy for the energy.

Fig. 5 shows one example of the correlation between the node coordination number and the internal energy. Fig. 5a shows the evolution of the internal energy and mean node coordination number for a simulation taken from Section 3.4.2, cooled over 100 μs. Fig. 5b further highlights the correlation between $U_{\mathrm{pair}}$ and 〈k〉, with scatter points being the positions of 〈k〉 and $U_{\mathrm{pair}}$ shown at different time points in Fig. 5a. Similar correlations exist across all the simulations we performed. The linear link between $U_{\mathrm{pair}}$ and 〈k〉 makes 〈k〉 useful, because it is independent of simulation parameters and smooths out thermal noise.

### 3.3 Energetics of idealised network structures

The actual network structures adopted in collageneous networks has been a topic of some discussion. Timpl et al. and more recently Cummings et al. [16], [34] favour a “chain-link fence” network, effectively a square-net structure. Burd [42], has alternatively suggested a primarily hexagonal network. Yurchenco and Furthmayr suggest a disordered collagen IV network interacting with an ordered scaffold [14], [43]. Imaging experiments have shed some light on the structure of the networks. However, complicating factors such as the biological origins of the networks, the presence of surfaces, or dissolved salts, mean that the matter has not been conclusively resolved.

**Fig. 6:**
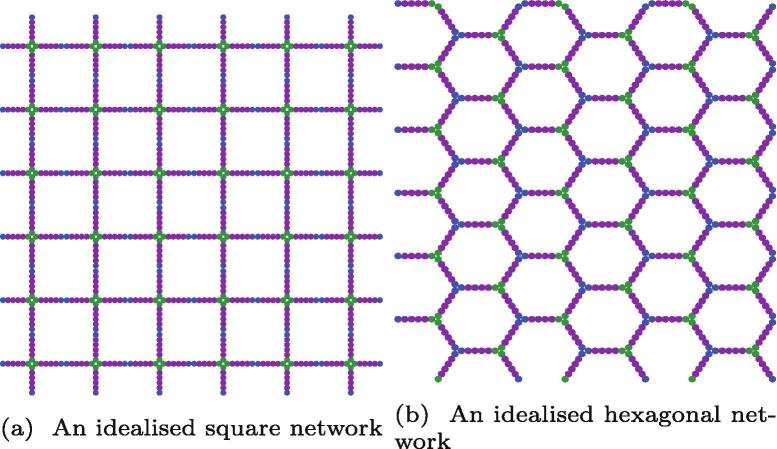
A comparison of two idealised networks, one square net (left panel — where each edge is two molecules long) akin to a chain-link fence, and hexagons akin to a honeycomb (right panel — where each edge is one molecule long).

Using our current polymer model, the energies of both square and hexagonal networks were compared. The initial networks are shown in Fig. 6a and Fig. 6b. The idealised networks were constructed at their energy minima (corresponding to all bonds remaining unstretched), and scaled in the *xy* plane. The stretching simulations revealed that the simple potential model used here always favours the hexagonal structure over the square net structure, regardless of the choice of $k_{l}$and ${∊}_{\mathrm{HH}}$. However, an improved potential model that better takes the preference of 7S and NC1 domains for different coordination numbers (k=2 and k=4 instead of k=3 for both types) may reverse this stabilisation order and favour a square net. Equally, environmental conditions or scaffolds may also reverse the stabilisation order.

Data on the stretching simulations can be found in Section SI IV of the Supplementary Information.

### 3.4 Control of the network structure

Having established a highly simplified model and metrics to describe the results, this work next investigates the sensitivity of network structure to the parameters of underlying coarse grained polymer model and simulation conditions.

#### 3.4.1 Polymer potential parameters

Collective behaviour, such as network assembly, can be strongly affected by the properties of the individual assembling units — here, the simplified model polymer. With any model, it is important to capture as much of the critical physics of a system while retaining computationally affordability. Two such important physical properties of collagen IV are how stiff or elastic a molecule is, because of the need for elasticity in the lens capsule, and the flexibility of the collagen IV molecule, which has been shown to be key in forming networks [30]. Here the impact of both the molecular stiffness, controlled by varying the energy scale of the harmonic bonds $k_{l}$, and the non-linear flexibility governed by changing the energy of the angular bonds $K_{\theta }$, are investigated, while all other parameters used are the defaults discussed in Section 2.3. There is a brief discussion of the effects of intrinsic curvature (by changing the equilibrium angle between three beads) in Section SI III, which was found to only have minor effects.

The bond strength was found to have little effect on the nature of the polygons formed, and the mean length of an edge in the network did not change with $k_{l}$, remaining at 〈l〉=317nm±53nm. This matches the spacing between interaction sites for untangled molecules observed by Yurchenco and Furthmayr [14], although it is longer than the distance between tangled sites in the more complex networks observed by Barnard et al. [44]. This indicates that polymers successfully forming a network do not deviate significantly from their equilibrium length in order to accommodate network formation. This near-invariance to $k_{l}$ allows the selection of convenient energy and timescales for simulations such that relatively long time-scale properties (such as network ageing) can be accessed while retaining relatively rigid polymers.

Changing the angular strength parameter, $K_{\theta }$ in the range $5\times 10^{-21}J⩽K_{\theta }⩽4\times 10^{-19}J$ had two major effects. First, it reduced the average length of an edge in the network from 295nm for “loose” angles (low $K_{\theta }$) to 319 nm (high $K_{\theta }$) for “stiff” angles. This is because the head-to–head distance within one molecule is shorter if the backbone curves, and looser molecules can bend more easily to accommodate better head group interactions. The longer edge lengths better reproduce the lengths observed in biological networks [34]. Second, it decreased the number of polygons successfully formed in the networks, from 81.0 on average in the loosest case to 66.0 on average in the stiffest case. This demonstrates that the bending of the polymers is important in order to allow network formation. These data are available in Table 1, and justify the importance of a worm-like curve model to network formation.

**Table 1:** The mean edge length 〈l〉, number of polygons $N_{\mathrm{polygon}}$, and the standard deviation of the number of polygons $\sigma_{N_{\mathrm{polygon}}}$ counted across 10 simulations as the angular strength $K_{\theta }$ changes.

$K_{\theta }$/ $1\times 10^{-21}$J	〈l〉/ nm	$N_{\mathrm{polygon}}$	$\sigma_{N_{\mathrm{polygon}}}$
5	294.62	79.2	8.64
50	311.65	78.3	4.76
100	314.64	71.1	6.06
150	316.96	68.2	5.83
200	318.27	71.6	4.48
250	318.11	69.0	7.04
300	318.43	69.9	7.58
400	318.51	66.0	3.65

#### 3.4.2 Dynamical cooling time

**Fig. 7:**
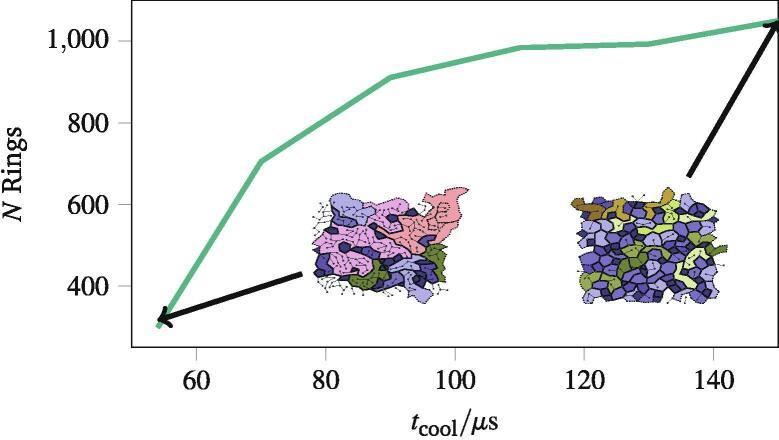
The number of polygons formed, *N*, against the time taken to cool the networks from 300Kto 30K, denoted $t_{\mathrm{cool}}$ with two networks cooled at different rates inset as examples. Different colours are used to highlight polygons with the same number of edges.

The timescales of self assembly are difficult to control in a laboratory setting, but relatively simple to control computationally. The freezing rate of the simplified polymers as they formed into networks was controlled by varying the time the polymers had to cool (referred to as $t_{\mathrm{cool}}$ in Section 2.3) from $t_{\mathrm{cool}}=54\;\mu s$ to $t_{\mathrm{cool}}=150\;\mu s$. When frozen rapidly, many fewer polygons were formed, varying from 31.6 polygons on average when cooled over $t_{\mathrm{cool}}=54\;\mu s$, to 105.0 on average when cooled over $t_{\mathrm{cool}}=150\;\mu s$. Fig. 7 shows the total number of polygons formed as a function of the cooling time, with all other parameters as discussed in Section 2.3. In addition, the figure shows two example networks to highlight the origin of the differences. The cooling solvent locks polymers into place before they can form full polygons, and many polymers end up “dangling”. As the cooling rate slows, the polymers have more time to slot into place in the evolving network.

When the polymer networks are cooled for longer, the average edge length and polygon areas remain similar — however, the average node coordination 〈k〉 increases to 2.996 for the slowest cooled network from 2.638 for the fastest cooled network. This can be inferred from Fig. 7, as the polygons in the fast-cooled networks feature more polygons with dangling edges, and a network with many fewer dangling edges in the slower-cooled network. The slow-cooled networks better resemble those seen in biology, and reinforce the value of a computational model simple enough to access long timescales.

#### 3.4.3 Molecular density

The huge diversity in the structures of biological networks is matched by a large diversity in network densities. To explore the effects of that diversity on the network structure, the molecule density (molecules per unit area) was varied whilst they were forming.

However, instead of assigning a potentially non-physical density *a priori*, we introduced an isothermal-isobaric (*NpT*) step in the simulation protocol, which allowed the periodic simulation cell size to be physically-determined rather than assigned based in relatively sparse data. After the simulation cell size was set in the *NpT*ensemble, it was thereafter fixed at the final value and the pressure coupling removed. The pressure in this section should not be interpreted directly, but instead as a proxy for the molecule density, for two reasons. The first is that nature of the simulation means that the position of particles are described by standard 3D Cartesian coordinates but images in the *z*direction are effectively infinitely separated. This means that pressure is a more convenient physical measure than stress, which would be strictly accurate for 2D systems. The second is that effects of the implicit solvent are neglected when performing the *NpT*step as the Langevin thermostat is disabled, and a Nosé-Hoover thermostat is applied in its place as described in Section 2.3.

**Fig. 8:**
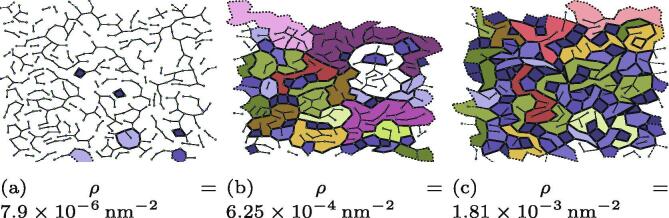
A comparison of three networks cooled at low, intermediate, and high molecule densities. Different colours are used to highlight polygons with the same number of edges, and these networks are drawn at different scales.

Below a pressure of p=2Pa at the lowest molecule densities no networks form. At low molecule densities the available volume for each molecule is so large that the mean intermolecular interaction energies are relatively small. As pressures increase, the coordination number per node increases and the average area and number of edges of polygons decreases. These data can be seen in Table 2, and examples are shown in Fig. 8. Networks formed at intermediate densities often had dangling edges, and networks at high densities had many small polygons forced together, surrounded by large polygons. The number of dangling edges is seen in Table 2, which counts the number of edges which are not involved in any polygon. For a full description of how the dangling nodes are quantified, see the polygon finding algorithm discussion in Bailey et al. [12].

**Table 2:** Average molecules per unit area 〈ρ〉, average coordination number 〈k〉, average edges per polygon 〈n〉, and the percentage of dangling edges (D.E.) which do not form parts of polygons, as a function of molecular density.

p/Pa	$\langle \rho \rangle /{\mathrm{nm}}^{-2}$	〈k〉	〈n〉	D.E. /%
5	$4.60\times 10^{-4}$	2.588	6.789	62.6
10	$6.25\times 10^{-4}$	2.691	7.039	47.5
20	$1.01\times 10^{-3}$	2.799	6.823	36.55
50	$1.81\times 10^{-3}$	3.062	6.034	23.875

#### 3.4.4 Lennard-Jones interaction strength

One well-studied aspect of collagen network formation is the effect of salts dissolved in the solvent, which can affect the stiffness of collagen molecules [45], the rate of network formation [16] and the structure of the network [15]. These different effects are difficult to deconvolute from one another experimentally; for example one cannot easily tell how the stiffness of monomers changes the final network without simultaneously changing the rate and energetics of network formation. In a simulation of self assembling polymers, these parameters are more easily separable and can be individually varied which can shed light on how multiple salting effects contribute.

**Fig. 9:**
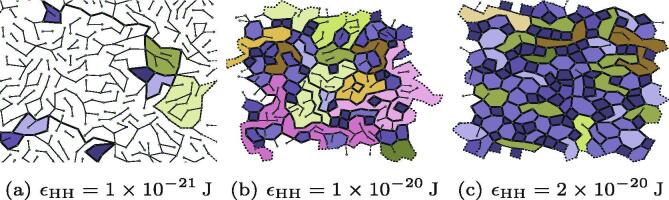
A comparison of three networks formed at low, intermediate, and high Lennard-Jones well depths. Polygons that have the same number of edges are coloured the same.

We tested the effect of energetics of head-group interaction by varying the well-depth for Lennard-Jones interactions in the range $1\times 10^{-21}J⩽{∊}_{\mathrm{HH}}⩽2\times 10^{-20}J$. Some example networks are shown in Fig. 9 at different values of ${∊}_{\mathrm{HH}}$, and highlight the dramatic effect on network structure. Relatively weak Lennard-Jones interactions lead to irregular networks that do not show any significant polygonal character. In this weak attraction regime there is sufficient thermal energy to disconnect nodes in the network for a longer period. This meant that the networks did not build up highly-coordinated sites, and remained as connected chains. Strong Lennard-Jones attractions encouraged more effective polygon network formation, with more hexagons and squares. The energetic reward for head groups interacting in this regime meant the average coordination number approached 3, and more small polygons were formed. Data on these networks are available in Table 3 which bear a significant resemblance to the results in Table 2. This is because both *p*and ${∊}_{\mathrm{HH}}$ affect the ratio between the kinetic energy of monomers and the potential energy gained in forming a network. By changing this ratio, the balance shifts between enthalpy guiding network formation towards a thermodynamically rewarding arrangement, and the kinetics locking a network into place as it forms. Studies of real collagen IV networks suggest that the coordination of 7S tetramers cannot be thermally reversed, and that thermal irreversibility is important to network formation [14].

**Table 3:** Average polygon area, average edges per polygon 〈n〉, average coordination number 〈k〉 of networks as a function of Lennard-Jones well depth.

${∊}_{\mathrm{HH}}/1\times 10^{-21}J$	$\langle \mathrm{Area}\rangle /{\mathrm{nm}}^{2}$	〈n〉	〈k〉
2	720321	6.422	2.69
4	386607	6.892	2.82
6	332060	6.923	2.87
8	282086	6.564	2.95
10	261117	6.409	2.98
12	257307	6.375	2.98
14	241528	6.273	3.01
16	231528	6.095	3.03
20	217023	5.925	3.05

#### 3.4.5 Pre-seeding lateral “Tangling” interactions

**Fig. 10:**
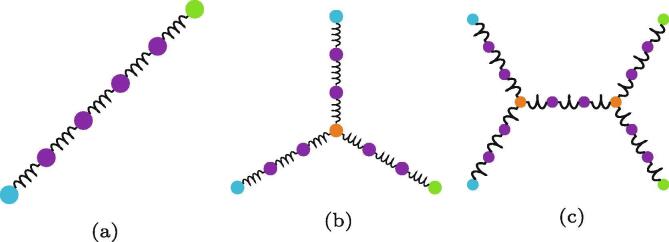
Three example molecules used in the simulations, demonstrating the ability to seed a simulation with pre-tangled interactions. Purple circles represent body beads, contributing to $U_{\mathrm{BB}}$, and green/ blue beads are head beads that contribute to $U_{\mathrm{HH}}$, similar to those shown in Fig. 6a. Finally, orange circles represent tangling sites (which can be seen in action in Fig. 11), but are otherwise identical to body beads. The gap from a blue/green head bead to an orange tangling site is 150nm, which matches a distance between interaction sites observed by Yurchenco and Furthmayr [14].

One reason biopolymers exhibit a richness in network structure is that their interactions are not limited to the terminal interactions of head groups. For example, as 3D objects, collagen IV molecules may tangle around one another. Yurchenco and Ruben argue that these lateral tangling interactions are key to forming an amorphous polygon network [43], [46]. In a 2D simulation it is possible to capture some of these 3D lateral interactions by pre-forming tangled molecules and introducing them to the network. Some example molecules are shown in Fig. 10, chosen as the simplest possible tangles that match observed images [14]. These are schematic molecules, with coloured circles representing beads that interact by Lennard-Jones interactions with one another and springs being harmonic potentials between beads in a molecule.

**Fig. 11:**
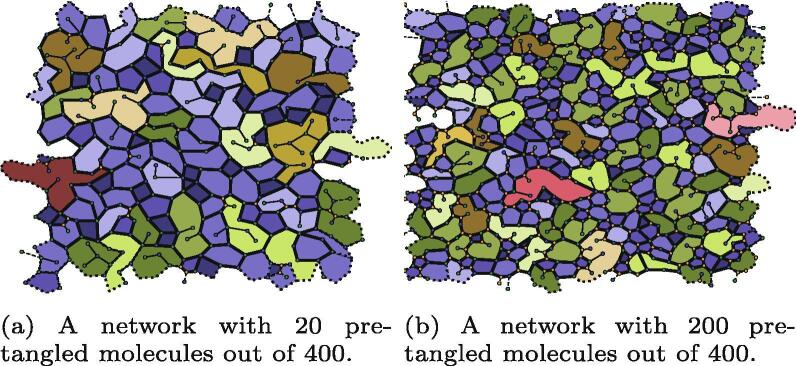
Comparison of networks formed with pre-tangled polymers. The nodes are coloured by the same scheme as used in Fig. 10.

The simulation procedure was followed as described above, but with between 0% and 50% of the linear polymers substituted for their tangled counterparts. Two example networks can be seen in Fig. 11, showing the propensity of tangling sites to lead to a greater number of small polygons. The addition of tangling sites also reduces the width of the *k*distribution, measured by $\mu_{2}(k)$, as each tangle point had a fixed value of k=3. Data on $\mu_{2}(k)$ can be seen in Table 4, which show a clear decrease in $\mu_{2}(k)$ as a greater percentage of straight polymers are replaced with tangled polymers. The Fig. 10c tangled molecules had a greater effect on $\mu_{2}(k)$ because they had two sites of fixed *k*instead of one or zero. The decreased $\mu_{2}(k)$ lead to a better fit to experimental networks, as discussed further in Section 3.5. The networks with different fractions of tangling sites had similar short-range orderings, as represented by the assortativity *r*in Table 4, showing a slight trend towards a more random short range order (with *r*closer to 0) when there are more tangling sites.

**Table 4:** Width of the node coordination distribution as measured by its second moment $\mu_{2}(k)$ for simulations in which a certain percentage of linear molecules have been replaced with tangled molecules seen in Fig. 10.

nFig. 10b/ %	$n_{\mathrm{Fig}.10c}/\%$	$\mu_{2}(k)$	*r*	$\langle \mathrm{Area}\rangle /{\mathrm{nm}}^{2}$
0	2.5	0.443	−0.116	245 473
0	25	0.257	−0.082	173 958
2.5	0	0.454	−0.114	247 026
2.5	2.5	0.417	−0.104	235 151
2.5	25	0.250	−0.068	203 734
25	0	0.281	−0.070	205 269
25	2.5	0.295	−0.084	190 625
25	25	0.215	−0.083	145 209

The average polygon area decreased in the networks with more tangling sites. This is to be expected, as the tangled molecules were not a one-to-one replacement for single polymers and had a greater density of head group sites. The presence of tangling sites could better capture the properties of physical networks and allow for a potential model with head group preferences that better reflects real networks.

### 3.5 The network landscape

The simulation results discussed here can be viewed in the context of Bailey et al. [12]. In that work the formation of disordered collagen-like networks was studied starting from a regular hexagonal network and systematically switching the bonds, maintaining an average value of 〈k〉, effectively modelling the network structure as entirely entropically-driven. The work also highlighted the role of the assortativity as an effective metric in characterising the network structure. Furthermore, the differences between networks could be best differentiated by considering a “network landscape” which shows the assortativity as a function of the width of the *k* distribution, as characterised by $\mu_{2}(k)$. While this technique better simulated 2D biological networks than previous bond switching techniques, it identified a gap where it was not possible to produce networks with a certain range of polygon assortativities *r*and distribution widths of *k*.

**Fig. 12:**
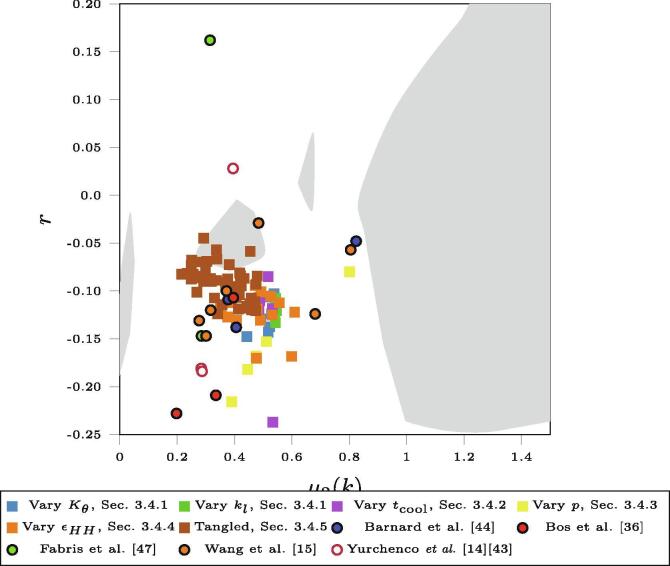
A “network landscape”, showing the assortativity *r*(representing short range order of polygons) against the second moment of the node coordination number distribution $\mu_{2}(k)$ (representing the range of node types). The simulated points in coloured squares match experimental data points (circles) better than previous work presented in Bailey et al. [12], which are represented as shaded regions.

Fig. 12 shows a “network landscape” similar to that in Bailey et al. [12], capturing *r*and $\mu_{2}(k)$ for the networks discussed in the current work. The shaded regions highlight the regions of the landscape accessible to the entropic models. The figure also shows the results obtained from analysis of experimental images (from Refs. [44], [36], [47], [15], [14], [43]). The values obtained from experiment show a wide range of values reflecting both the different conditions under which images are obtained and the difficulties in extracting detailed information. Experimental images occupy regions of the network landscape excluded from the entropically-driven models, which had studied the effects of temperature, cooling rates, and limits on *k*. Fig. 12 shows the results from the present work, varying the system variables as discussed above.

Each set of simulations discussed earlier is present as a different coloured set of squares, where each square represents the result of a single simulation. Critically, the introduction of controlled inter- and intra-molecular interactions (enthalpic contributions) generates network configurations which significantly fill the regions of the network landscape occupied by the experimental configurations. The majority of parameter changes result in a clear relationship between *r* and $\mu_{2}(k)$ with the former becoming less negative (less dissassortative) as $\mu_{2}(k)$ increases. This is most obvious in the results from Section 3.4.2 and Section 3.4.3, and also holds for the results from Section 3.4.1. This relationship can be explained by reference to the assortativity of polygons formed by a random point process, which is r≈-0.15 [20]. This disassortativity for an entirely random process comes from geometrical constraints; with *k*and 〈n〉fixed, small polygons must border large polygons [12]. When a range of values is allowed for *k*(that is, $\mu_{2}(k)\;\neq \;0$), these constraints no longer hold and the system can approach a more random organisation of polygons with r≈0. Varying the Lennard-Jones parameter ${∊}_{\mathrm{HH}}$ as in Section 3.4.4 and the number of tangled nodes as in Section 3.4.5 produces a more complicated behaviour. The simulations with tangled interactions lead to a cluster of points on the landscape with changing $\mu_{2}(k)$ but relatively similar *r*, demonstrating the control that is possible over simulated networks by changing the starting molecules. The effects of ${∊}_{\mathrm{HH}}$ lead to a similar clustering of landscape points in the region r≈-0.10 and $0.25⩽\mu_{2}(k)⩽0.6$. This is because the energetics of network formation leave the short-range order relatively unaffected.

## 4 Conclusion

In conclusion, a highly simplified model for collagen IV has been constructed which is shown to self assemble to form well-defined biological networks. The “worm-like” polymer model features physically meaningful parameters that allow control over the networks formed. The polymer networks are significantly different from typical inorganic networks, for example easily forming convex, irregular polygon structures. There is a rich range of networks that can be formed, since varying simple parameters changes the balance between enthalpy (guiding the shape of polygons), entropy (affecting the ordering of polygons) and kinetics (locking networks into shape as they form).

The properties of the polymers themselves can affect the networks that are formed. The worm-like curve model allows for polymer flexibility, which encourages network formation as polymers can deform out of position to better coordinate into a network. The rate of cooling, external pressure and strength of interaction are all important in controlling the nature of the final networks. By varying key variables it has been demonstrated that a key factor in network formation is the kinetics of node-forming events where two head groups encounter one another to create a node in the network (which is thermally irreversible) and locked forming networks into place.

Finally, the networks formed in this work have filled in a gap established in previous work in the network landscape, and again highlight the critical balance of enthalpic, entropic and kinetic factors.

## CRediT authorship contribution statement

**Matthew H.J. Bailey:** Data curation, Investigation, Software, Visualization, Writing - original draft. **Mark Wilson:** Conceptualization, Project administration, Supervision, Writing - review & editing.

## Declaration of Competing Interest

The authors declare that they have no known competing financial interests or personal relationships that could have appeared to influence the work reported in this paper.
