# Supplementary material for: Self assembly of model polymers into biological random networks
Source: Comput Struct Biotechnol J. 2021 Feb 12;19:1253–62. doi: 10.1016/j.csbj.2021.02.001 (PMC7918283; doi:10.1016/j.csbj.2021.02.001)
Supplement: Supplementary file 1 [file mmc1.pdf]

Self Assembly of Biological Random Networks Electronic Supplementary Information†

Matthew H J Bailey,* and Mark Wilson*

SI I Clustering Algorithm.

The atomic positions are converted into a graph representation in the following algorithmic steps:

- Find all pairs head groups within a cutoff radius, here chosen to be $r = \sqrt[6]{2}\sigma_{HH}$ where σ_{HH} is the Lennard-Jones radius.
- Find all connected components of this pair list. If there are pairs $(1,2)$ and $(2,3)$, form the component $1,2,3$.
- Make each connected component a node in a new graph, with the position being the mean average position of its head group members.
- Connect components with an edge if there is a path between members of two different components along molecule bonds.
  - If there are "tangling sites" along a molecule, or multiple paths, follow the shortest path between the components and represent it as multiple edges in the final graph, split at the "tangling sites"
- Follow the polygon finder algorithm as detailed in the Electronic Supplementary Information of Bailey *et al.* [1]

SI II Shape Regularity Criterion

Full data for the shape regularity criterion of Section 3.2 are shown in Table SI 1. The silica data from Kumar *et al.* [2] are in the range 0.957 to 0.967, whilst images of biological networks are in the range 0.693 to 0.911.

Data from simulations are available in Table SI 2. These are in the range 0.77 to 0.95 depending on the simulated parameter.

SI III Equilibrium Angles.

Polymers can often have intrinsic curvature in solution, and this may be an important property for network formation. To investigate whether intrinsic curvature had any effect on the collagen-like model molecules, we set the equilibrium angle between two beads in the polymer to θ_{eqm} in the range 120° to 180°.

For $\theta_{\mathrm{eqm}} = 120°$, the molecules had no net curvature. This is because both left-handed and right-handed angles were populated approximately evenly along the chains, as depicted in Figure SI 1.

For molecules with $130° \leq \theta_{eqm} \leq 160°$, there was a small amount of polymer curvature. The polymers could not swap from

Name	Figure	$\langle SRC \rangle$	σ_{SRC}
Kumar *et al.* [2]	2a	0.966	0.024
Kumar *et al.* [2]	2b	0.964	0.028
Kumar *et al.* [2]	2c	0.957	0.032
Kumar *et al.* [2]	2d	0.962	0.025
Kumar *et al.* [2]	2e	0.967	0.023
Barnard *et al.* [3]	2	0.792	0.147
Barnard *et al.* [3]	4c	0.832	0.115
Barnard *et al.* [3]	6	0.848	0.113
Bos *et al.* [4]	2a (alternative)	0.853	0.146
Bos *et al.* [4]	2a	0.834	0.123
Bos *et al.* [4]	5c	0.836	0.103
Bos *et al.* [4]	5d	0.854	0.097
Fabris *et al.* [5]	3f	0.837	0.114
Fabris *et al.* [5]	4b	0.810	0.128
Wang *et al.* [6]	1c	0.781	0.159
Wang *et al.* [6]	2a	0.810	0.145
Wang *et al.* [6]	2d	0.841	0.106
Wang *et al.* [6]	3b alternative	0.693	0.218
Wang *et al.* [6]	3b	0.830	0.133
Wang *et al.* [6]	4a	0.847	0.128
Wang *et al.* [6]	5b	0.770	0.151
Wang *et al.* [6]	5c	0.847	0.107
Yurchenco and Furthmayr [7]	4b alternative	0.874	0.092
Yurchenco and Furthmayr [7]	4b	0.848	0.105
Yurchenco and Ruben [8]	1c	0.871	0.088
Yurchenco and Ruben [8]	2f	0.911	0.081

Table SI 1 Comparison of regularity data between Kumar *et al.* [2] and simulated networks cooled at different rates, showing that slower-cooled networks lead to more regular polygons. The simulated data matches the regularity metrics of polygons in images of collagen networks better than it matches silica networks. See Bailey *et al.* [1] for more details on how the experimental data were obtained.

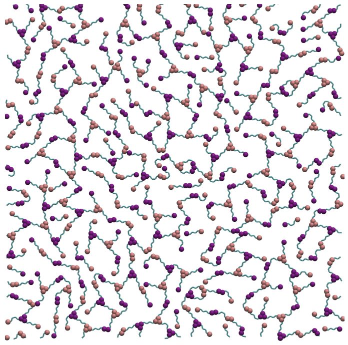

Fig. SI 1 A simulation run with $\theta_{\mathrm{eqm}} = 120°$. The molecules end up with little net curvature, as the angles cancel out with one another akin to a skeletal hydrocarbon.

Department of Chemistry, Physical and Theoretical Chemistry Laboratory, University of Oxford, South Parks Road, Oxford OX1 3QZ; E-mail: mark.wilson@chem.ox.ac.uk

† Electronic Supplementary Information (ESI) available.

* these authors contributed equally to this work.

Parameter	Value	Unit	$\langle SRC \rangle$	σ_{SRC}
θ_{eqm}	120	°	0.769	0.174
θ_{eqm}	130	°	0.854	0.113
θ_{eqm}	140	°	0.809	0.147
θ_{eqm}	150	°	0.746	0.193
θ_{eqm}	160	°	0.771	0.192
θ_{eqm}	170	°	0.788	0.181
θ_{eqm}	180	°	0.799	0.185
K_θ	5.0×10^{-20}J		0.763	0.179
K_θ	1.0×10^{-19}J		0.782	0.186
K_θ	1.5×10^{-19}J		0.783	0.176
K_θ	2.0×10^{-19}J		0.788	0.181
K_θ	2.5×10^{-19}J		0.800	0.182
K_θ	3.0×10^{-19}J		0.794	0.182
K_θ	4.0×10^{-19}J		0.786	0.177
K_θ	5.0×10^{-19}J		0.774	0.185
k_l	2.0×10^{-5}	$\mathrm{N\,m^{-1}}$	0.790	0.177
k_l	5.0×10^{-5}	$\mathrm{N\,m^{-1}}$	0.801	0.174
k_l	1.0×10^{-4}	$\mathrm{N\,m^{-1}}$	0.787	0.186
k_l	1.5×10^{-4}	$\mathrm{N\,m^{-1}}$	0.790	0.177
k_l	2.5×10^{-4}	$\mathrm{N\,m^{-1}}$	0.788	0.178
t_{cool}	54	μs	0.806	0.158
t_{cool}	70	μs	0.781	0.184
t_{cool}	90	μs	0.805	0.174
t_{cool}	110	μs	0.822	0.166
t_{cool}	130	μs	0.828	0.162
t_{cool}	150	μs	0.831	0.159
ε_{HH}	1.0×10^{-21}J		0.844	0.114
ε_{HH}	1.5×10^{-21}J		0.796	0.161
ε_{HH}	2.0×10^{-21}J		0.769	0.179
ε_{HH}	2.5×10^{-21}J		0.748	0.194
ε_{HH}	3.0×10^{-21}J		0.770	0.185
ε_{HH}	3.5×10^{-21}J		0.772	0.189
ε_{HH}	4.0×10^{-21}J		0.780	0.185
ε_{HH}	4.5×10^{-21}J		0.796	0.181
ε_{HH}	5.0×10^{-21}J		0.801	0.176
ε_{HH}	5.5×10^{-21}J		0.798	0.178
ε_{HH}	6.0×10^{-21}J		0.810	0.168
ε_{HH}	8.0×10^{-21}J		0.835	0.157
ε_{HH}	1.0×10^{-20}J		0.839	0.154
ε_{HH}	1.2×10^{-20}J		0.855	0.141
ε_{HH}	1.4×10^{-20}J		0.860	0.134
ε_{HH}	1.6×10^{-20}J		0.857	0.140
ε_{HH}	2.0×10^{-20}J		0.866	0.138
p	0.2	Pa	0.950	0.0553
p	0.5	Pa	0.928	0.0893
p	2	Pa	0.871	0.142
p	5	Pa	0.823	0.171
p	20	Pa	0.787	0.187
p	50	Pa	0.781	0.154

Table SI 2 *SRC* data for all simulations discussed in this work.

a left-handed angle to a right-handed angle, and when a molecule was created with net curvature it then could not straighten up. This meant that the polymers were shorter and often curled up in critical locations to prevent network formation.

For molecules with $160° \leq \theta_{eqm} \leq 180°$, the polymers again formed networks. These networks featured shorter edges, as the polymers could switch zig-zag orientations to straighten themselves out. The ring metrics for these close-to-straight polymers are similar to those for straight polymers.

SI IV Idealised Network Structures Data

The energies as a function of area per molecule are shown in Figure SI 2 for a sample simulation with $\varepsilon_{HH} = 4.142\times10^{-21}$ J and $k_l = 1.657\times10^{-4}\,\mathrm{N\,m^{-1}}$, and all other parameters are the defaults as discussed in Section 2.3 of the main paper. The stretching simulations are independent of angular parameters, as all molecules are straight.

Firstly, these graphs show that the hexagonal network is more densely packed than the square-net network. This is contrary to the densities of square-nets and hexagonal networks in inorganic chemistry (where hexagonal networks are less dense), as each square in the square-net has a perimeter of 8 molecules, instead of a perimeter of 6 molecules for the hexagons. These show that the hexagonal networks are more energetically stable at an area of $86\times10^3\,\mathrm{nm}^2$ per molecule, and are destabilised primarily due to bond stretching as the area per molecule increases. Only when significantly stretched above $107\times10^3\,\mathrm{nm}^2$ per molecule does the square net become energetically favourable, as the molecules are less densely packed. The square net is metastable with an energetic minimum at an area of $118\times10^3\,\mathrm{nm}^2$ per molecule. A transition from a hexagonal network to a square net is kinetically unfavourable, as a large concerted rearrangement would be necessary to move all coordination sites from $k = 2$ and $k = 4$ to $k = 3$. The presence of a laminin scaffold, or surface on which the network rests, in a real network would make this transition even more difficult.

The exact energy measures of this stretching are linearly dependent on the choice of k_l and ε_{HH}, the energy scales of harmonic bonds and head group interactions. The simple model used here stabilises a hexagonal network at all k_l and ε_{HH} values.

Notes and references

1 M. H. J. Bailey, D. Ormrod Morley and M. Wilson, *RSC Adv.*, 2020, **10**, 38275–38280.

2 A. Kumar, D. Sherrington, M. Wilson and M. F. Thorpe, *J. Phys. Condens. Matter*, 2014, **26**, 1–6.

3 K. Barnard, S. A. Burgess, D. A. Carter and D. M. Woolley, *J. Struct. Biol.*, 1992, **108**, 6–13.

4 K. J. Bos, D. F. Holmes, R. S. Meadows, K. E. Kadler, D. McLeod and P. N. Bishop, *Micron*, 2001, **32**, 301–306.

5 G. Fabris, A. Lucantonio, N. Hampe, E. Noetzel, B. Hoffmann, A. DeSimone and R. Merkel, *Biophys. J.*, 2018, **115**, 1770–1782.

6 Z. Wang, Q. Xiao, X. Song, Y. Wan and J. Zhu, *J. Food Qual.*, 2017, **2017**, 1–10.

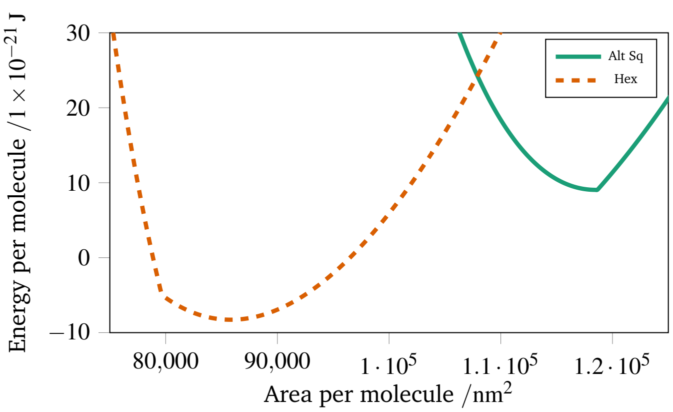

Fig. SI 2 A comparison of two idealised networks, one made of hexagons akin to a honeycomb and one made of squares (where each edge is two molecules) akin to chickenwire. The networks were scaled isotropically with scale factor $\lambda = 0.9$ to 1.2. This was converted into an area per molecule as the networks had different initial areas.

7 P. D. Yurchenco and H. Furthmayr, *Biochemistry*, 1984, **23**, 1839–1850.

8 P. D. Yurchenco and G. C. Ruben, *J. Cell Biol.*, 1987, **105**, 2559–2568.

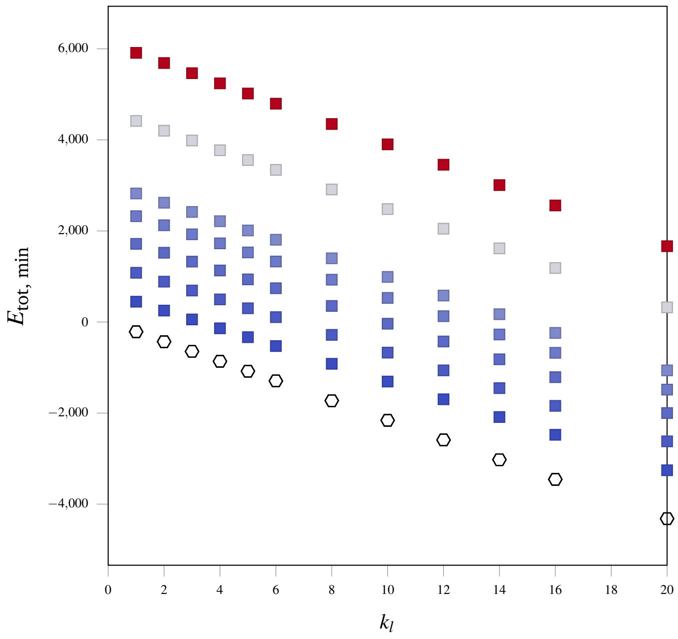

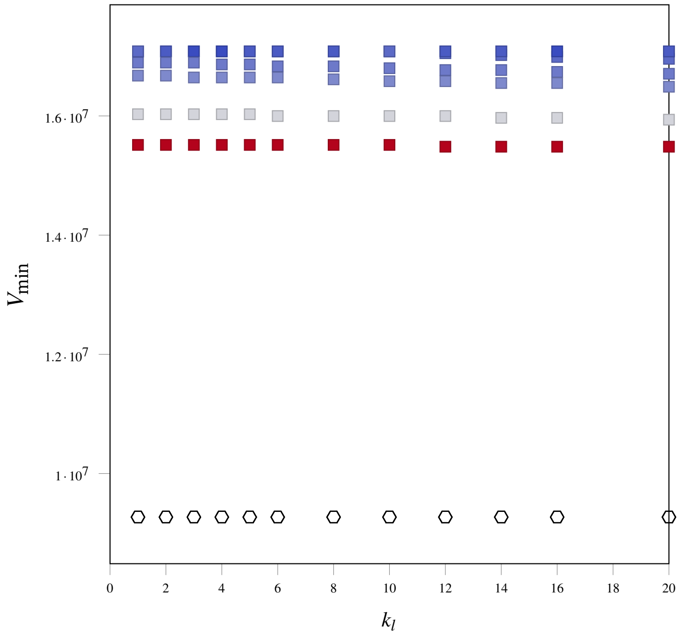

Fig. SI 3 Hexagons represent the energy of the minimum for the hexagonal net when k_l and ε_{HH} are changed. V_{min}. ε_{HH} is a very minor factor, so $E_{\text{tot, min}}$ is just linear in k_l. Squares represent square nets, and they are separated vertically by ε_{HH} which affects them more — a redder colour indicates a larger ε_{HH}, and a bluer colour represents a lower ε_{HH}. However, the square nets are still dominated by k_l and are always more positive in energy than the hexagonal net.
